# 

**Published:** 1919-12-27

**Authors:** 


### THE HOSPITAL

**The Modern Newspaper of Administrative Medicine and Institutional Life.**

Telegraphic Address: OSPEDALE, RAND, LONDON. Telephone Number: 2734 GERRARD

No. 1751, Vol. LXVII. | Saturday, December 27th, 1919 | PRICE TWOPENCE

